# Supplementary material for: Modeling glioblastoma heterogeneity as a dynamic network of cell states
Source: Mol Syst Biol. 2021 Sep 16;17(9):e10105. doi: 10.15252/msb.202010105 (PMC8444284; doi:10.15252/msb.202010105)
Supplement: Supplementary file 6 — Source Data for Figure 5 [file MSB-17-e10105-s004.zip › Figure5A_sourcedata/GSEA_3071/hallmarks_stateVI.GseaPreranked.1623742557031/HALLMARK_ESTROGEN_RESPONSE_EARLY.html]

Details for gene set HALLMARK\_ESTROGEN\_RESPONSE\_EARLY[GSEA]

|  || Dataset | state63071 |
| Phenotype | NoPhenotypeAvailable |
| Upregulated in class | na\_pos |
| GeneSet | HALLMARK\_ESTROGEN\_RESPONSE\_EARLY |
| Enrichment Score (ES) | 0.2052706 |
| Normalized Enrichment Score (NES) | 1.3613784 |
| Nominal p-value | 0.08227848 |
| FDR q-value | 0.13917711 |
| FWER p-Value | 0.591 |
Table: GSEA Results Summary

  

Fig 1: Enrichment plot: HALLMARK\_ESTROGEN\_RESPONSE\_EARLY      
 Profile of the Running ES Score & Positions of GeneSet Members on the Rank Ordered List

  

| PROBE | GENE SYMBOL | GENE\_TITLE | RANK IN GENE LIST | RANK METRIC SCORE | RUNNING ES | CORE ENRICHMENT || 1 | TGM2 |  |  | 3 | 1.367 | 0.0425 | Yes |
| 2 | KRT8 |  |  | 42 | 0.895 | 0.0602 | Yes |
| 3 | MYC |  |  | 69 | 0.815 | 0.0788 | Yes |
| 4 | PRSS23 |  |  | 108 | 0.732 | 0.0914 | Yes |
| 5 | CALB2 |  |  | 116 | 0.711 | 0.1119 | Yes |
| 6 | FLNB |  |  | 120 | 0.709 | 0.1336 | Yes |
| 7 | UGCG |  |  | 124 | 0.700 | 0.1549 | Yes |
| 8 | DLC1 |  |  | 129 | 0.696 | 0.1758 | Yes |
| 9 | MYOF |  |  | 157 | 0.655 | 0.1890 | Yes |
| 10 | PMAIP1 |  |  | 255 | 0.561 | 0.1797 | Yes |
| 11 | FKBP4 |  |  | 279 | 0.550 | 0.1907 | Yes |
| 12 | XBP1 |  |  | 389 | 0.494 | 0.1760 | Yes |
| 13 | FHL2 |  |  | 397 | 0.491 | 0.1896 | Yes |
| 14 | MAST4 |  |  | 446 | 0.473 | 0.1912 | Yes |
| 15 | WWC1 |  |  | 495 | 0.456 | 0.1922 | Yes |
| 16 | KLF10 |  |  | 506 | 0.453 | 0.2038 | Yes |
| 17 | IGFBP4 |  |  | 571 | 0.433 | 0.1996 | Yes |
| 18 | PAPSS2 |  |  | 600 | 0.425 | 0.2053 | Yes |
| 19 | OLFML3 |  |  | 721 | 0.392 | 0.1842 | No |
| 20 | FOXC1 |  |  | 927 | 0.357 | 0.1382 | No |
| 21 | MYBBP1A |  |  | 980 | 0.349 | 0.1348 | No |
| 22 | PPIF |  |  | 983 | 0.348 | 0.1452 | No |
| 23 | SLC7A5 |  |  | 989 | 0.348 | 0.1549 | No |
| 24 | RRP12 |  |  | 1217 | 0.313 | 0.1014 | No |
| 25 | SLC7A2 |  |  | 1347 | 0.298 | 0.0748 | No |
| 26 | MLPH |  |  | 1373 | 0.295 | 0.0772 | No |
| 27 | DYNLT3 |  |  | 1472 | 0.283 | 0.0588 | No |
| 28 | FDFT1 |  |  | 1552 | 0.274 | 0.0454 | No |
| 29 | KRT18 |  |  | 1570 | 0.272 | 0.0493 | No |
| 30 | SLC2A1 |  |  | 1606 | 0.268 | 0.0480 | No |
| 31 | AR |  |  | 1810 | -0.251 | -0.0008 | No |
| 32 | MAPT |  |  | 1922 | -0.267 | -0.0233 | No |
| 33 | SVIL |  |  | 1939 | -0.269 | -0.0192 | No |
| 34 | NCOR2 |  |  | 2134 | -0.303 | -0.0638 | No |
| 35 | TIAM1 |  |  | 2303 | -0.334 | -0.1001 | No |
| 36 | ABAT |  |  | 2438 | -0.365 | -0.1260 | No |
| 37 | SLC39A6 |  |  | 2474 | -0.371 | -0.1240 | No |
| 38 | GFRA1 |  |  | 2540 | -0.387 | -0.1299 | No |
| 39 | GREB1 |  |  | 2576 | -0.393 | -0.1273 | No |
| 40 | IGF1R |  |  | 2618 | -0.404 | -0.1259 | No |
| 41 | TFF3 |  |  | 2670 | -0.416 | -0.1270 | No |
| 42 | RPS6KA2 |  |  | 2709 | -0.428 | -0.1240 | No |
| 43 | RAB31 |  |  | 2806 | -0.459 | -0.1363 | No |
| 44 | OLFM1 |  |  | 2875 | -0.485 | -0.1399 | No |
| 45 | ADCY1 |  |  | 2882 | -0.488 | -0.1261 | No |
| 46 | SLC26A2 |  |  | 2883 | -0.489 | -0.1106 | No |
| 47 | PDLIM3 |  |  | 3011 | -0.546 | -0.1288 | No |
| 48 | RETREG1 |  |  | 3057 | -0.566 | -0.1234 | No |
| 49 | CELSR2 |  |  | 3121 | -0.598 | -0.1220 | No |
| 50 | RBBP8 |  |  | 3189 | -0.652 | -0.1201 | No |
| 51 | FARP1 |  |  | 3275 | -0.734 | -0.1205 | No |
| 52 | BCL11B |  |  | 3276 | -0.735 | -0.0972 | No |
| 53 | MPPED2 |  |  | 3283 | -0.741 | -0.0754 | No |
| 54 | RHOBTB3 |  |  | 3400 | -0.893 | -0.0795 | No |
| 55 | CHPT1 |  |  | 3470 | -1.048 | -0.0656 | No |
| 56 | FOS |  |  | 3476 | -1.056 | -0.0335 | No |
| 57 | TUBB2B |  |  | 3532 | -1.228 | -0.0100 | No |
| 58 | NBL1 |  |  | 3535 | -1.240 | 0.0288 | No |
Table: GSEA details [plain text format]

  

Fig 2: HALLMARK\_ESTROGEN\_RESPONSE\_EARLY: Random ES distribution      
 Gene set null distribution of ES for **HALLMARK\_ESTROGEN\_RESPONSE\_EARLY**
